# Accuracy and reliability of artery–vein differentiation in small-field macular OCT angiography

**DOI:** 10.1117/1.JMI.13.2.025501

**Published:** 2026-03-20

**Authors:** Haneen Alfauri, Tugce Ilayda Turer, Cyriac Manjaly, Aditya Santoki, Senyue Hao, Marin Woronets, Chao Zhou, Rithwick Rajagopal

**Affiliations:** a Washington University in Saint Louis, Department of Electrical & Systems Engineering, St. Louis, Missouri, United States; b Washington University School of Medicine, Department of Ophthalmology & Visual Sciences, St. Louis, Missouri, United States; c Washington University in Saint Louis, Department of Biomedical Engineering, St. Louis, Missouri, United States

**Keywords:** artery–vein differentiation, optical coherence tomography angiography, small-field optical coherence tomography angiography, macular optical coherence tomography angiography

## Abstract

**Purpose:**

Accurate artery–vein (AV) differentiation in small-field macular optical coherence tomography angiography (OCTA) remains challenging due to a lack of standardized guidelines. We propose and validate criteria for 3 × 3 mm^2^ (10 deg × 10 deg on Spectralis; ~2.9 × 2.9 mm^2^) macular scans.

**Approach:**

Small field-of-view (FOV) OCTA scans were analyzed using established AV criteria for large-field (12 × 12 mm^2^) OCTA, as applied by two masked readers and validated against color fundus photographs (CFPs) and near-infrared reflectance (NIR) images. Accuracy and reliability (Cohen’s *κ*) were assessed. Pixel-level AV masks were annotated with a standardized threshold. Vessel diameters and intensities were compared within our dataset and in the publicly available OCTA-500 dataset to assess whether intrinsic vessel features support AV differentiation.

**Results:**

A total of 465 vessels from 20 healthy eyes were evaluated across 3 pseudo-branching orders using the criteria for OCTA. Annotators achieved high accuracy (95.1%, 92.3%) and strong intra/inter-rater reliability (*κ* = 0.84) with similarly high AV classification accuracy within pseudo-third-order vessels (97.15%). No significant AV diameter differences were observed in either dataset (*p* = 0.261 and 0.442). The mean intensity was similar in our dataset (*p* = 0.277; ∣Δ∣ = 3.28, 1.45% relative difference) but higher for veins in OCTA-500 (*p* < 0.0001; ∣Δ∣ = 3.42, 1.63% relative difference).

**Conclusions:**

Accurate and reproducible AV labeling is feasible in 3 × 3 mm^2^ scans, with strong inter- and intra-rater agreement. Vessel diameter and intensity add limited value. NIR-based alignment of OCTA with CFP provides reliable ground truth, supporting consistent manual labeling and OCTA segmentation.

## 1 Introduction

Optical coherence tomography angiography (OCTA) is a non-invasive imaging modality that offers high-resolution, 3D visualization of retinal blood vessels using decorrelation among sequential B-scans at the same location.^1-3^ Recent advances in OCTA have sparked interest in artery–vein (AV) differentiation, which has potential utility in clinical assessment of retinal vascular health and as an investigational tool.^4,5^ For example, AV differentiation in the macular region may enhance our understanding of vessel-specific pathology in common blinding conditions such as diabetic retinopathy (DR), central retinal vein occlusion (RVO), and hypertensive retinopathy.^6-10^

Small-field OCTA (3 × 3 mm^2^ to 6 × 6 mm^2^) offers superior detail and remains the clinical standard for its high resolution in detecting localized issues (e.g., venous dilation and arterial narrowing). Although wide-field (12 × 12 mm^2^) offers broader coverage but with a lower spatial density (resolution), AV mapping adds diagnostic value but is challenging in small-field OCTA due to limited landmarks and poor alignment with color fundus photos (CFPs). Ishibazawa et al.^11^ demonstrated reliable AV differentiation in wide-field (12 × 12 mm^2^) scans using vessels near the optic nerve, which are absent in smaller fields (3 × 3 mm^2^ and 6 × 6 mm^2^). These scans are fovea-centered, further complicating gold-standard CFP mapping.

This study addresses the lack of standardized guidelines for AV differentiation in small-field OCTA. We formalize OCTA-specific criteria and assess AV labeling accuracy and reliability in a 3 × 3 mm^2^ macular field of view (FOV). To overcome limited FOV challenges, we introduce a near-infrared reflectance (NIR) image mapping strategy linking high-resolution OCTA with gold-standard CFP. Focusing on this clinically relevant region, we highlight the strengths and limitations of macular OCTA for reliable AV differentiation.

## 2 Methods

### 2.1 Study Population

Healthy adult volunteers were recruited at the Department of Ophthalmology, Washington University in St. Louis. Participants were required to be over 18 years of age and able to provide informed consent. Individuals with a history of ocular surgery, retinal pathology, or systemic vascular disease were excluded. All participants had normal ocular health at the time of examination. The study was approved by the Institutional Review Board at Washington University in St. Louis and conducted in accordance with the Declaration of Helsinki.

### 2.2 Imaging Datasets and Acquisition Parameters

Foveal-centered OCTA images were acquired using a 10 deg ×10 deg scan angle on the Heidelberg Spectralis OCTA system (Heidelberg Engineering, Heidelberg, Germany).^12^ On the retina, this corresponds to ~2.9 × 2.9 mm^2^. For consistency with common clinical nomenclature, we refer to this acquisition as a nominal “3 × 3 mm^2^” scan throughout the paper. The Spectralis system uses spectral-domain technology with a 880-nm wavelength and 85-kHz A-scan rate.^12^ Each scan included 512 A-scans × 512 B-scans, yielding an isotropic lateral resolution of 5.7 *μ*m/pixel and axial resolution of ~3.9 *μ*m/pixel in tissue, enabling visualization of fine retinal capillaries with a sampling density

(1) $$\text{Spatial resolution (sampling density)}=\frac{512\;\text{Ascans}}{2.9\;\text{mm}}\approx 177\;\text{Ascans}∕\text{mm}.$$

OCTA images were generated using full-spectrum amplitude decorrelation angiography (FSADA), a probabilistic method producing high-contrast, nearly binarized images from four repeated B-scans. *En face* images of the superficial retinal layer (internal limiting membrane to inner plexiform layer) were exported. Trained technicians performed imaging, repeating scans as needed to ensure signal strength ≥30 dB (per Heidelberg’s quality index) and minimal motion artifacts. Grading (annotation) was performed separately by non-physician study personnel who were trained by a retinal physician with experience in retinal image analysis. Moreover, the graders followed a standardized AV labeling protocol approved by the retinal physician. CFP images were acquired using the Optos Silverstone, a high-resolution, ultra-wide-field device (200-deg field of view) considered the gold standard for vessel identification. To supplement our dataset, we also utilized the OCTA-500 public dataset,^13^ a well-established resource containing expert-annotated *en face* OCTA images.

### 2.3 Vessel Selection for Annotation

**Fig. 1:**
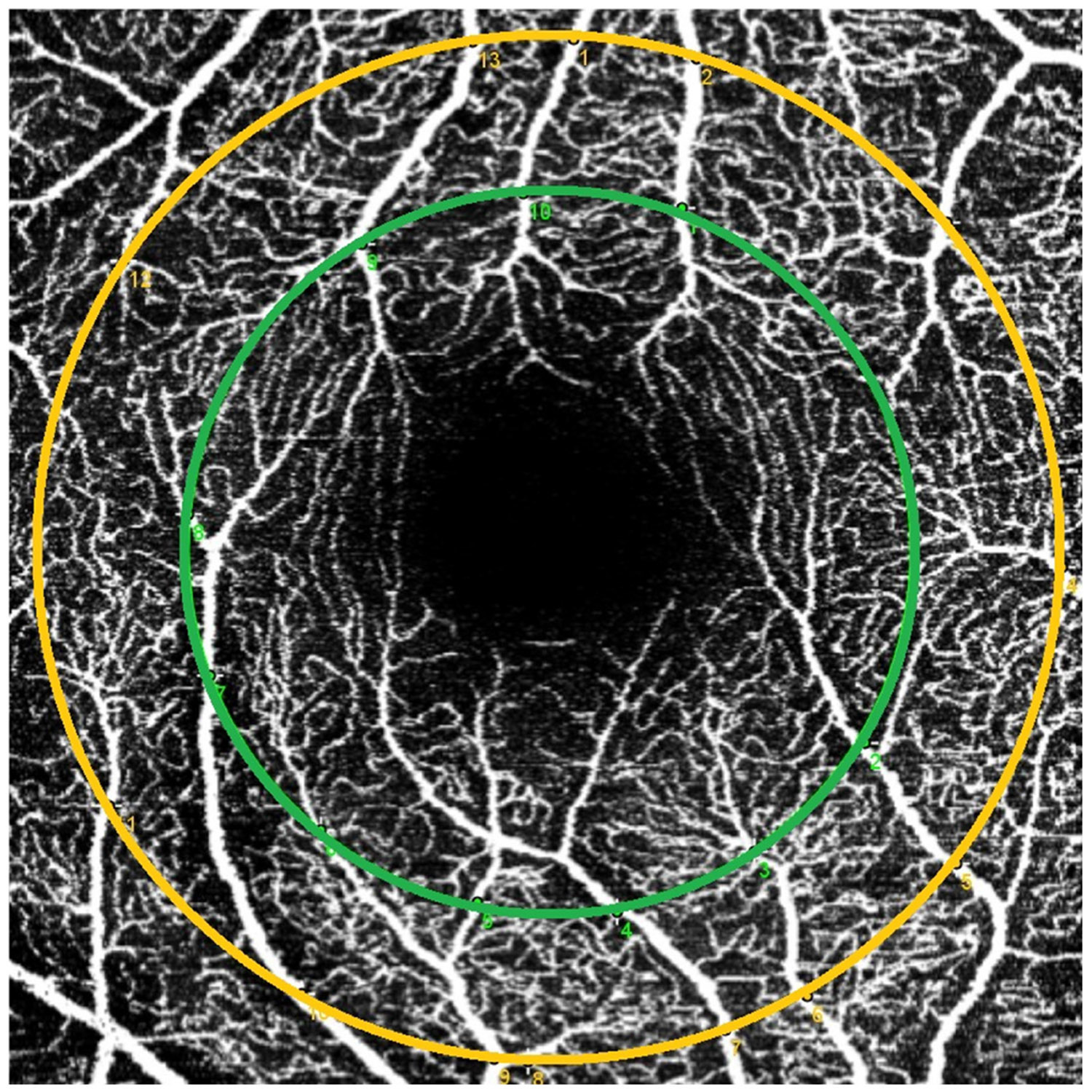
OCTA image with two concentric rings centered on the fovea: green (2.0-mm diameter, parafovea) and orange (2.75-mm diameter, scan edge). Used to standardize vessel selection across images.

To standardize vessel selection on each 3 × 3 mm^2^ OCTA image, we used two arbitrary concentric rings centered on the fovea to approximate anatomical regions of interest within the OCTA image. The green ring, with a diameter of 2 mm, was chosen to delineate the parafoveal zone. The orange ring, with a diameter of 2.75 mm, represents a boundary approaching the outer limit of the 3 × 3 mm^2^ FOV, as shown in Fig. 1.

Although these rings do not perfectly align with ETDRS or EMM5 standards (which typically extend to 3 to 6 mm for perifovea), they provide a scalable approximation suitable for analyses within the constraints of our 3 × 3 mm^2^ scan area.

Vessels intersecting these circles were graded as arteries or veins. Only pseudo-first-, second-, and third-order vessels were evaluated; “pseudo” indicates that classification was limited to the macular region, where a pseudo-first-order vessel is the largest segment within the field of view before branching, followed by its subsequent branches. In this context, the term “pseudo” refers to an approximate classification of vessel branching order based on relative size and appearance within the macular field of view.

We clarify that pseudo-branching order represents a local, within-field hierarchy and does not reflect true disc-traced anatomic vessel generation. Because 3 × 3 mm^2^ OCTA does not include the optic disc and therefore lacks upstream vascular context, branching order was intentionally defined locally within the macular field of view (“pseudo-branching order”), consistent with our goal of evaluating AV differentiation using small-field *en face* OCTA alone.

Accordingly, pseudo-first-, second-, and third-order vessels were defined based on their relative caliber and local branching pattern within the scan to standardize vessel selection and segment definition. Thus, pseudo-branching order served only as a within-field descriptor for organizing vessel hierarchy in the limited 3 × 3 mm^2^ macular OCTA scans. It was independently assigned by two graders, and any disagreements were adjudicated by a clinician to ensure consistency.

Two masked readers independently labeled vessels on *en face* 3 × 3 mm^2^ OCTA images without access to corresponding CFP or NIR. Intra- and inter-rater reliability were assessed by repeating grading at two time points (*T*1 and *T*2) ~2 weeks apart, with re-randomization and masking to prior labels to minimize recall bias. Annotators were trained study personnel with experience in retinal image analysis and underwent structured training on representative cases using a standardized AV labeling protocol.

### 2.4 Establishing Ground Truth

Mapping vessels in 3 × 3 mm^2^ *en face* OCTA images to CFP is challenging due to the differences in field of view and imaging modality, where detailed AV differentiation is needed but global spatial references are limited. To address this, we introduce an NIR-mediated mapping strategy. Commercial OCTA systems often include NIR confocal scanning laser ophthalmoscopy, which assists with scan alignment and produces images inherently co-registered with OCTA, typically covering a wider field of view.

In this study, co-registered NIR images were exported for the same region of interest to bridge the spatial gap between macular OCTA and wide-field CFP. This extended anatomical context enabled accurate vessel correspondence across modalities. Using this approach, we successfully transferred vessel labels from gold-standard CFP to the restricted macular OCTA images. To our knowledge, this is the first study to implement NIR-guided CFP vessel mapping to establish ground truth in small-field macular OCTA imaging. To establish artery and vein ground truth in CFP, a trained retinal physician (R. Rajagopal) manually traced vessels from the optic disc, where vascular structures are most distinguishable. The arteries were identified by their lighter red hue and thinner caliber, reflecting more light due to oxygenated blood,^14^ whereas veins appeared darker and slightly wider.

**Fig. 2:**
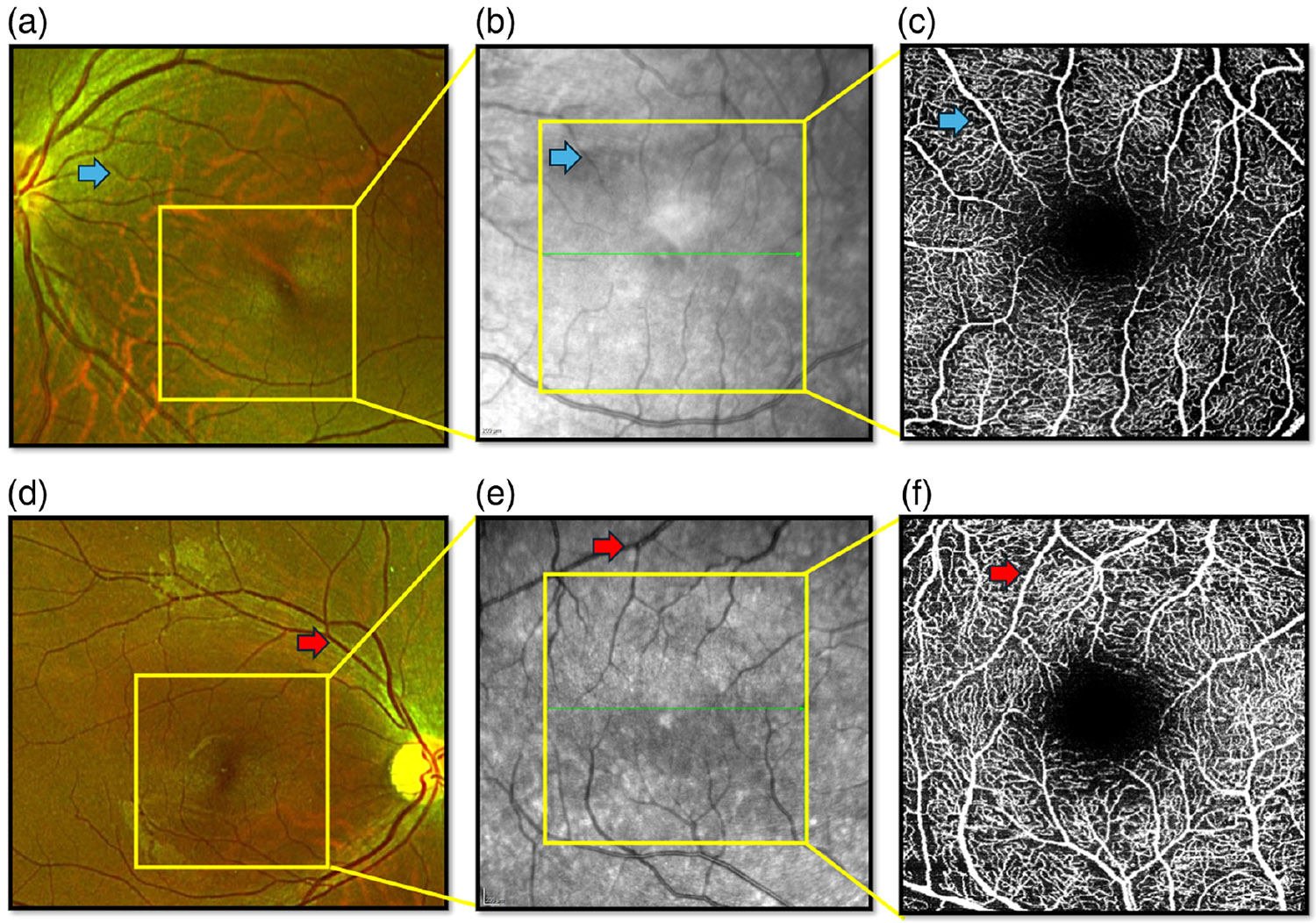
Ground truth mapping via multimodal alignment. (a) and (d) Wide-field CFP (200-deg FOV) cropped. (b) and (e) NIR (15-deg FOV). (c) and (f) OCTA (10-deg FOV). NIR acts as an intermediate step to enable accurate vessel registration between OCTA and CFP. Representative vessels were traced across modalities to establish correspondence: a vein in the top row [panels (a)–(c); blue arrow] and an artery in the bottom row [panels (d)–(f); red arrow].

Differentiation followed standard vascular anatomy and branching patterns. Once labeled at the disc, vessel identities were propagated distally. These CFP-labeled vessels served as the gold standard and were mapped to macular OCTA images using the corresponding NIR image. Figure 2 illustrates the three-step mapping process from wide-field (200 deg) CFP to 10 deg (3 × 3 mm^2^) OCTA via an intermediate 15-deg NIR image. After obtaining ground truth labels from CFP images, pixel-wise annotations were performed using GIMP (GNU Image Manipulation Program, version 2.10.38),^15^ an open-source image editing tool. A standardized “select by threshold” function was used to ensure consistent intra-image thresholding for accurate vessel boundary delineation. Separate binary masks were generated for the arteries and veins. These masks were then used to compare vessel diameter and intensity.

### 2.5 Statistical Analysis

Annotation accuracy was calculated by comparing each annotator’s labels on OCTA images to the gold-standard labels derived from CFP. Accuracy was defined as the percentage of vessels correctly labeled.

Annotation reliability was assessed using Cohen’s kappa coefficient (*κ*) for both inter- and intra-rater agreement. For inter-rater analysis, *κ* measured the agreement between two independent annotators, with the expected chance agreement calculated by multiplying the class proportions (artery and vein) of annotators A and B. For intra-rater analysis, *κ* was computed using the class proportions from a single annotator across two time points (*T*1 and *T*2).

To assess whether intrinsic vessel features could support AV classification, we first established ground truth labels for arteries and veins. Annotators then used the GIMP^15^ software to create pixel-level vessel masks, applying a consistent threshold to delineate vessel edges and ensure annotation consistency. These masks were subsequently used to compare vessel diameter and intensity between arteries and veins.

Vessel diameter was quantified through skeletonization of the vessels followed by a Euclidean distance transform, and intensity was measured as the average grayscale pixel value within each annotated region. Statistical comparisons were performed using the Wilcoxon signed-rank test, a non-parametric test suitable for paired, non-normally distributed data. All *p*-values were two-sided, and values less than 0.001 were considered statistically significant.

All analyses were conducted in Python using Google Colab. Cohen’s *κ* was computed using the cohen_kappa_score function from sklearn.metrics, and Wilcoxon tests were performed using scipy.stats.

### 2.6 Criteria for AV Differentiation in the Macula

Two independent, masked annotators without access to ground truth CFP or NIR images labeled the superficial *en face* OCTA images using the following criteria, when discernible. A brief background on each criterion is provided below.

**Fig. 3:**
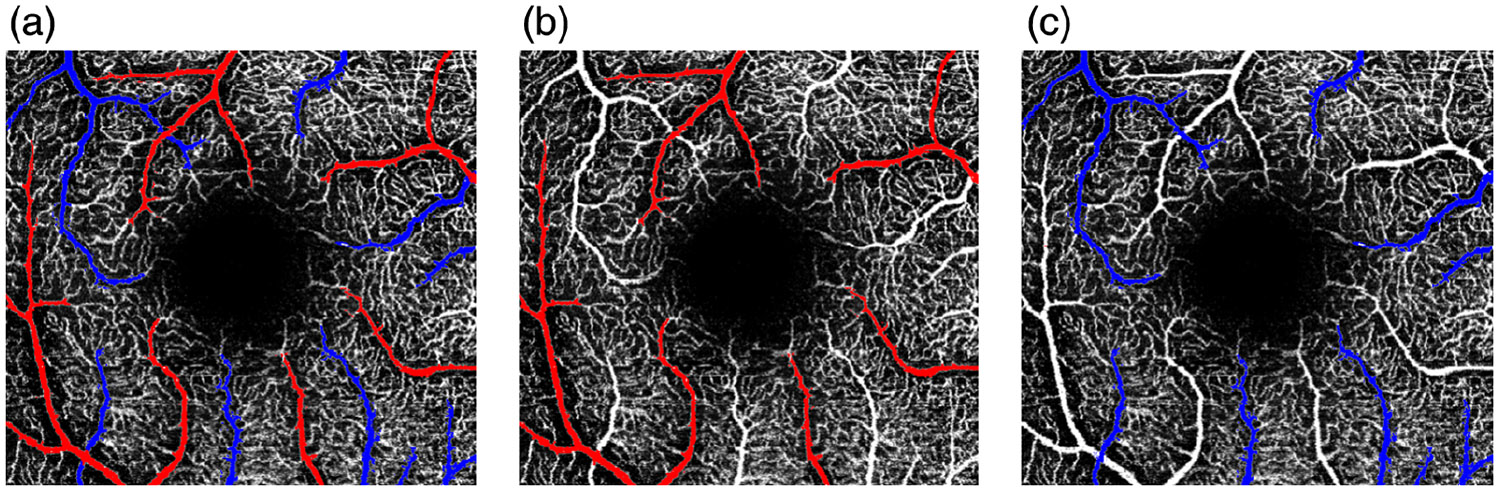
Annotated OCTA images showing vessel segmentation. (a) Arteries (red) and veins (blue). (b) Arteries only. (c) Veins only. A prominent CFZ surrounds the arteries, serving as a spatial reference for artery–vein differentiation in 3 × 3 mm^2^ scans.

**Fig. 4:**
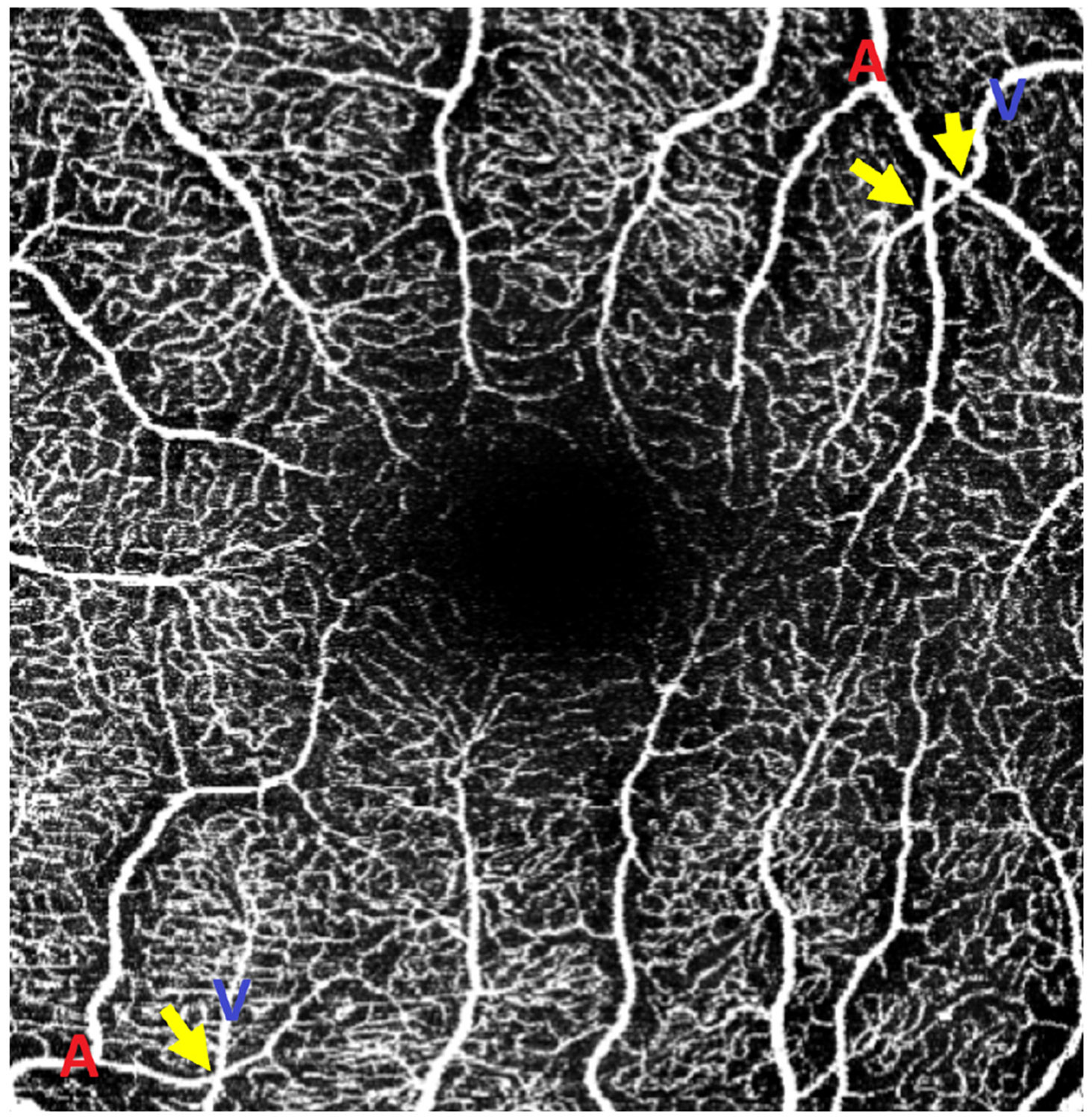
Annotated OCTA image illustrating arteriovenous (AV) crossings in the macular region. Yellow arrows indicate two crossing points where an artery (A) and a vein (V) intersect, labeled in red and blue, respectively. The visibility of arterial CFZ in addition to the crossing points provides additional anatomical context that aids in accurate artery–vein differentiation, especially in 3 × 3 mm^2^ scans.

- Capillary-free zone: The capillary-free zone (CFZ), or periarterial CFZ (paCFZ), is typically observed around the arteries but not the veins.^16,17^ It is thought to arise during embryogenesis due to oxygen diffusion from arterial walls, which elevates local pO_2_ and inhibits nearby capillary growth.^18^ Consequently, the arteries are often surrounded by hypointense halos, whereas the veins embedded in denser capillary networks lack such zones.^19^ Figure 3 shows prominent CFZs around arteries (red) and their absence around veins (blue), a distinction that remains visible in 3 × 3 mm^2^ macular OCTA.
- Vessel crossings: The arteries rarely cross arteries, and the veins rarely cross veins; most crossings occur between the arteries and the veins.^20^ This pattern can be a helpful cue for differentiation, especially when other features are unclear. Figure 4 shows an AV crossing in the macular region; when combined with the presence of a CFZ, it enables immediate AV differentiation.
- Vessel caliber: The arteries typically have smaller diameters than the veins, with differences varying by retinal location.^21-23^
- Vessel intensity: This refers to pixel brightness in OCTA images, reflecting the strength of the flow-related decorrelation signal. Intensity depends on acquisition settings (e.g., interscan time) and post-processing/decorrelation algorithms. Its use for manual AV differentiation has not been quantitatively validated in the literature for 3 × 3 mm^2^ macular OCTA images.
- Vessel tortuosity: This measures how much a vessel curves or deviates from a straight path, reflecting vascular integrity. It is assessed along segments between bifurcations or endpoints.

Table 1 summarizes the morphological features used for artery and vein differentiation, along with their applicability and visibility in macular OCTA images.

## 3 Results

The analysis was conducted on 20 healthy eyes from 20 adult volunteers (11 males and 9 females; mean age: 33 years, range: 22 to 54).

### 3.1 Accuracy Analysis

Table 2 summarizes the accuracy of AV differentiation across pseudo-first-, second-, and third-order vessels within a 3 × 3 mm^2^ macular field of view. Annotations from both annotators were compared against gold-standard labels established by an expert reader using CFP, serving as the ground truth. Accuracy reflects AV differentiation only and does not include agreement in pseudo-branching order. Pseudo-branching order was used solely as a within-field descriptor for standardized vessel selection in the limited 3 × 3 mm^2^ OCTA scan.

Accuracies 1 and 2 correspond to AV classification accuracy at the first (*T*1) and second (*T*2) grading sessions, respectively, for each annotator. Overall, both annotators achieved high accuracy across all vessel types, demonstrating the effectiveness of the proposed annotation guidelines in facilitating consistent labeling. Annotator 1 achieved an average overall accuracy of 95.1%, with error counts ranging from 0 to 23 across the different vessel orders. Annotator 2 also showed robust performance, with an overall average accuracy of 92.3% and a similar pattern of strong differentiation across vessel orders. Specifically, for pseudo-first-order vessels (315 total), annotator 1 achieved an average accuracy of 94.8%, whereas annotator 2 reached 92.9%. For pseudo-second-order vessels (115 total), the average accuracy was 94.3% for annotator 1 and 90.4% for annotator 2. Notably, performance was highest for pseudo-third-order AV differentiation (35 total), with annotator 1 achieving perfect accuracy (100%) and annotator 2 achieving 94.3%. Given the modest sample size of pseudo-third-order vessels (*N* = 35), this finding likely reflects dataset-specific sampling variability rather than indicating that pseudo-third-order AV differentiation is inherently easier.

### 3.2 Reliability Analysis

**Fig. 5:**
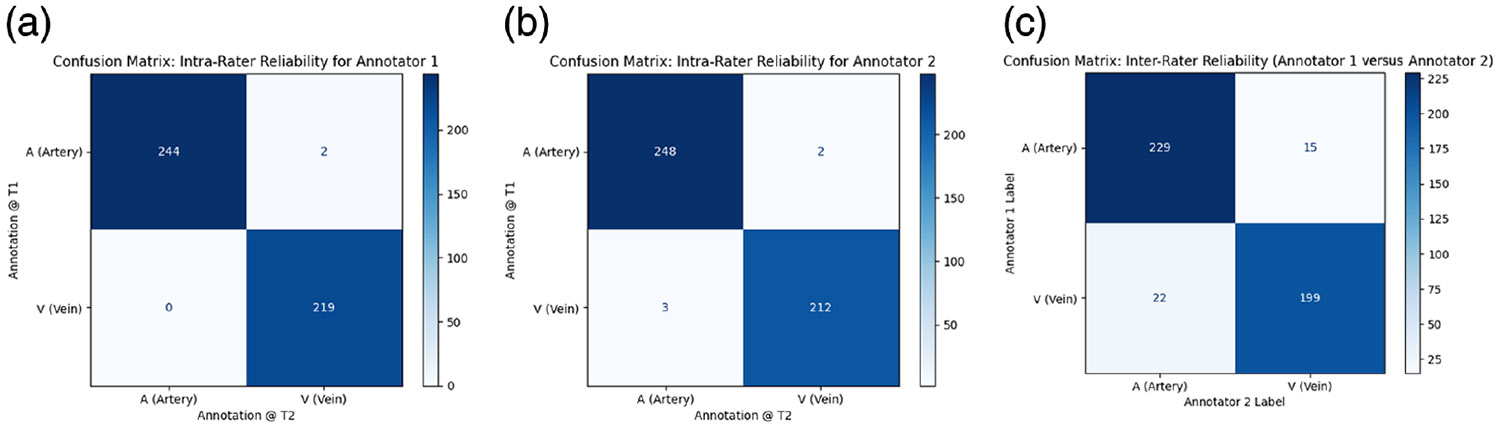
Confusion matrices quantifying (a) annotator 1’s intra-rater reliability (*κ* = 0.99), (b) annotator 2’s intra-rater reliability (*κ* = 0.98), and (c) inter-rater concordance.

Both annotators demonstrated strong intra-rater consistency across two annotation time points (*T*1 and *T*2). As shown in the confusion matrices, Figs. 5(a) and 5(b), annotator 1 showed only two directional disagreements (artery at *T*1 labeled as vein at *T*2) and no vein-to-artery disagreements, whereas annotator 2 showed two artery-to-vein and three vein-to-artery directional disagreements across sessions. Cohen’s *κ* values further support this high consistency, with annotator 1 achieving *κ* = 0.99 and annotator 2 achieving *κ* = 0.98 (Table 3). Inter-rater agreement was assessed by comparing the first set of annotations from both annotators.

Figure 5(c) demonstrates strong inter-rater agreement, with only 15 vessels labeled as artery by annotator 1 but vein by annotator 2, and 22 vessels labeled as vein by annotator 1 but artery by annotator 2. However, the overall inter-rater reliability remained high, with a Cohen’s *κ* of 0.84 as shown in Table 3, reflecting substantial agreement. Consistent *κ* values across pseudo-first-, second-, and third-order vessels (0.82, 0.84, and 0.80, respectively) further demonstrate the effectiveness of the structured annotation guidelines in supporting reliable AV differentiation within the small macular field of view.

### 3.3 OCTA Imaging Feature Analysis

**Fig. 6:**
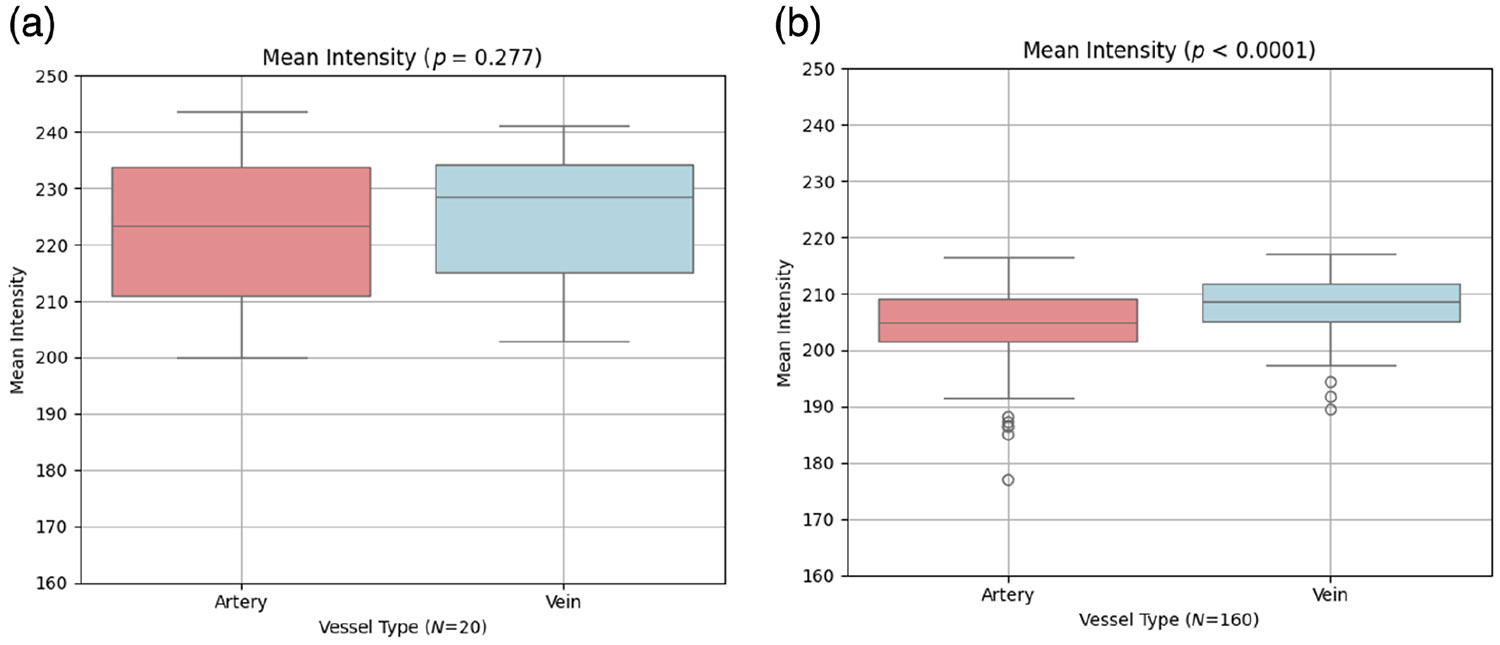
Comparison of the mean vessel intensity between the arteries and veins. (a) Our dataset (*N* = 20) shows a non-significant trend toward higher venous intensity (*p* = 0.277). (b) OCTA-500 dataset (*N* = 160) shows significantly brighter veins (*p* < 0.0001), likely due to the larger sample size.

**Fig. 7:**
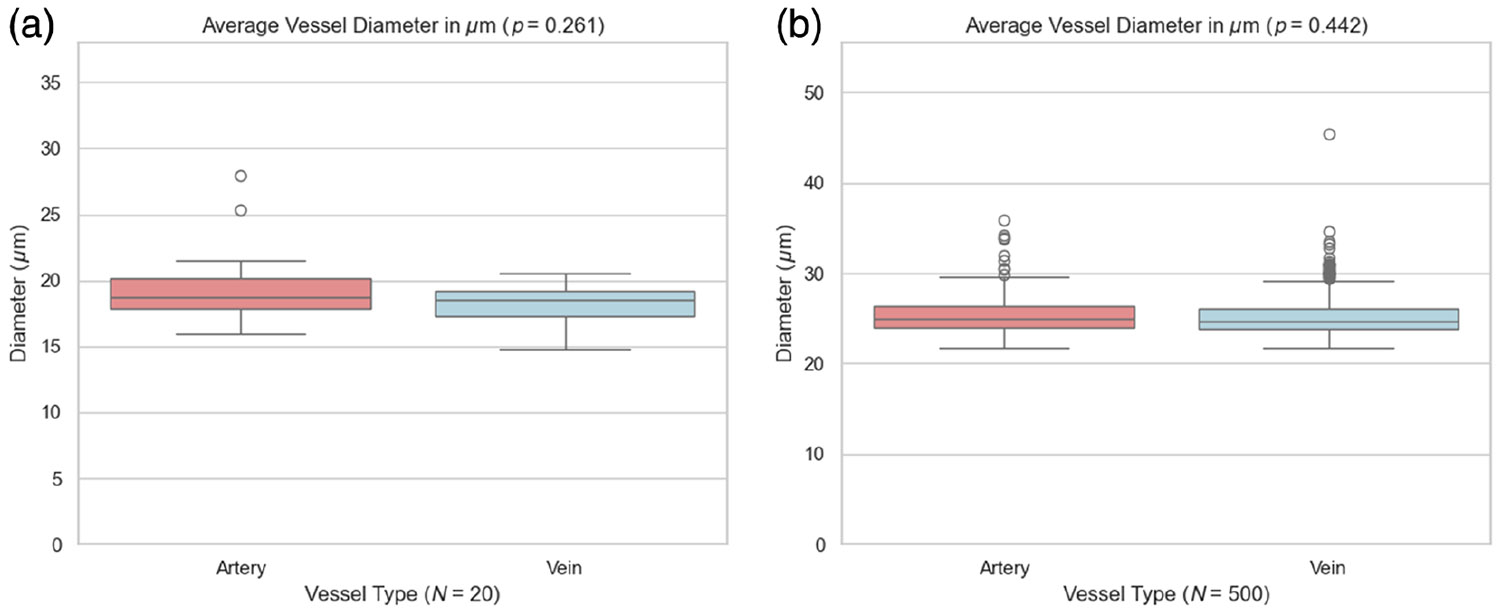
Comparison of the average vessel diameter between arteries and veins. (a) Our dataset (*N* = 20 eyes) shows no statistically significant difference in vessel diameter between arteries and veins (*p* = 0.261). (b) Similarly, the OCTA-500 3 × 3 mm^2^ dataset (*N* = 160 healthy eyes) also shows no significant difference in diameter among vessel types (*p* = 0.442). These findings suggest that vessel diameter alone may not be a reliable distinguishing feature for artery–vein classification in macular OCTA images. Absolute diameter values are not intended for cross-dataset biological comparison due to device- and processing-dependent variability and slight differences in FOV.

- Intensity analysis: Although faster flow is often assumed to increase OCTA signal intensity,^24,25^ OCTA brightness depends on acquisition parameters (e.g., interscan time) and decorrelation-based processing and may saturate at higher velocities. In our dataset, there was no difference in intensity between arteries and veins (*p* = 0.277). We observed a small magnitude increase in intensity of veins over arteries in the OCTA-500 dataset^13^ of 160 healthy eyes (*p* < 0.0001). However, the absolute intensity difference was small in both datasets: ∣Δ∣ = 3.28 (1.45%) in ours and ∣Δ∣ = 3.42 (1.63%) in OCTA-500, as shown in Fig. 6.
- Vessel caliber analysis: We quantitatively compared mean arterial and venous diameters in 3 × 3 mm^2^ macular OCTA scans using both our dataset and the OCTA-500 dataset.^13^ As shown in Fig. 7, neither dataset showed a statistically significant artery–vein diameter difference (*p* = 0.261 and *p* = 0.442, respectively). In our dataset, artery and vein diameters were 19.4 ± 2.8 and 18.3 ± 1.3 *μ*m, respectively. In OCTA-500, artery and vein diameters were 25.3 ± 1.9 and 25.2 ± 2.1 *μ*m, respectively. Although absolute diameter estimates differ among datasets, these values are not intended for cross-dataset biological comparison because OCTA-derived diameter measurements can vary with device-specific sampling and segmentation/processing methodology and can also be affected by slight differences in scan field-of-view (our 2.9 × 2.9 mm^2^ scans, denoted as 3 × 3 mm^2^, versus the true 3 × 3 mm^2^ OCTA-500 scans). Accordingly, we restrict interpretation to within-dataset artery–vein comparisons and do not draw cross-dataset conclusions from absolute diameter values.

## 4 Discussion

This study demonstrates the feasibility of AV differentiation in small-field macular OCTA scans. Our results establish a baseline accuracy of 93.7% (mean across annotators) for normal vessel differentiation, which will support more meaningful interpretation in future studies involving diseased retinas. Our findings are comparable to those reported by Ishibazawa et al.^11^ who used 12 × 12 mm^2^ wide-field OCTA (98.61% accuracy with Cohen’s *κ* = 0.948), using 3 × 3 mm^2^ scans; annotator 1 demonstrated near-perfect intra-rater reliability (Cohen’s *κ* = 0.99), and annotator 2 showed similarly high consistency (*κ* = 0.98). Inter-rater agreement was substantial (*κ* = 0.84). One important limitation of our study is that the exclusive focus on healthy eyes may overestimate classification accuracy relative to pathological conditions, where features such as capillary dropout could obscure key structural cues, particularly the paCFZ.

The small 3 × 3 mm^2^ field of view presents two related constraints: it captures fewer complex arteriovenous crossings (limiting evaluation of the “crossing artifact” noted in wider-field studies) and excludes larger first-order vessels. Paradoxically, this restricted view also serves as a strength, as it simplifies the validation framework by reducing confounding variables while concentrating on the diagnostically critical macular microvasculature. Although wide-field OCTA emphasizes global vessel architecture, small FOV scans provide significantly higher spatial resolution, enabling clearer visualization of microvascular structures. This enhanced spatial detail is particularly important for detecting subtle pathological changes, such as arterial narrowing and venous dilation, which are often missed in broader scans. However, because the optic disc lies outside the 3 × 3 mm^2^ field of view, pseudo-branching order represents a within-field hierarchy and may not correspond to true disc-traced vascular generation. Accordingly, pseudo-branching order was used only to standardize vessel selection and define vessel segments within the macular scan, not to support conclusions requiring global disc-centered vascular topology. Clinically, this limitation is unlikely to have much significance. In current practice, small-field OCTA without the optic nerve head is widely used. Within these small fields, assignment of the vessel type (artery or vein) carries greater clinical relevance than the assignment of correct branching order.

Importantly, our findings show that high AV differentiation accuracy is achievable even for small, challenging vessels, which is particularly valuable for applications in early disease detection and monitoring. Reliability analyses further support the reproducibility and feasibility of differentiating arteries and veins within the limited macular field of view. Although AV differentiation may become more challenging in more distal branches due to reduced caliber and weaker intensity cues, we observed the highest accuracy in pseudo-third-order vessels in this dataset. Because this subgroup included only 35 vessels, this result should be interpreted cautiously and likely reflects sampling variability and dataset-specific factors (e.g., scan quality and visibility of paCFZ and branching cues), rather than indicating that higher-order (more distal) vessel classification is inherently easier.

Beyond manual annotations, we quantitatively evaluated traditional morphological features commonly cited in the literature for AV differentiation. Notably, vessel diameter, long considered a reliable indicator, showed no statistically significant difference between arteries and veins in either our dataset or the OCTA-500 dataset within the macular 3 × 3 mm^2^ FOV. This confirms that vessel caliber differences diminish at the macula region and reinforces that in small-FOV OCTA scans (where pseudo-first and higher-order vessels dominate), diameter is not a dependable feature for AV differentiation. Similarly, although the AV crossing rule is highly reliable near the optic disc, it remains applicable though less frequently observed in macular regions. Exceptions are rare and typically arise under pathological conditions. Nonetheless, the rule generally holds in healthy retinal anatomy.

Tortuosity, although elevated in disease states such as DR or RVO, showed no significant artery–vein differences in healthy maculae using 9 × 9 mm^2^ FOV (Gao et al.^22^). In small FOVs, the reliability of tortuosity measurements is limited by short vessel segments and a lack of curvature context.

Moreover, paCFZ size varies with retinal oxygenation, which can be altered in vascular pathologies such as DR and RVO.^26,27^ Thus, the paCFZ serves not only as a reliable morphological cue for artery–vein differentiation but also as a potential non-invasive biomarker for retinal oxygenation. This feature holds clinical value and presents a promising spatial indicator for training automated models to distinguish arteries from veins in OCTA images.

Vessel intensity analysis in our dataset showed no significant difference between the arteries and the veins (*p* = 0.277, ∣Δ∣ = 3.28, 1.45% relative difference) where the percentage reflects the difference relative to the vein intensity for each pair. In contrast OCTA-500 dataset (*N* = 160, 3 × 3 mm^2^ FOV) showed slightly higher vein intensity (*p* < 0.0001, ∣Δ∣ = 3.42, 1.63% relative difference), also calculated with respect to the corresponding vein value. The statistical discrepancy likely stems from differences in sample size (*N* = 20 versus 160), image quality, and OCTA processing methods. Importantly, the datasets were acquired on different platforms: our scans used the Heidelberg Spectralis with FSADA, whereas OCTA-500 used the RTVue-XR Avanti (Optovue Inc., Fremont, California, United States) with split-spectrum amplitude decorrelation angiography.^3^ Variations in scanning speed, wavelength, and motion contrast algorithms likely contributed to the observed intensity differences. This raises important questions about the fidelity of flow-based signal representation and underscores the need to validate OCTA-derived vascular metrics across imaging platforms. Device-specific acquisition settings and processing algorithms can influence intensity profiles and must be considered when interpreting vessel intensity and flow-related measurements.

Tomographic sectioning is a key advantage of OCT, and cross-sectional structural B-scans and flow-overlaid B-scans may provide additional anatomic cues for AV differentiation, particularly in challenging cases such as vessel overlap or arteriovenous crossings.^11,20^ In this work, however, our primary objective was to establish standardized criteria for AV differentiation using small-field (3 × 3 mm^2^) macular *en face* OCTA, which is the format most commonly exported and used in clinical and machine-learning workflows. Accordingly, readers were masked to B-scans and other modalities during grading to evaluate AV classification. Incorporating depth-resolved reflectance features and flow-overlaid B-scans represents an important future direction to further enhance robustness, particularly in diseased eyes and at complex crossing sites.

## 5 Conclusion

This study addresses a key limitation in macular OCTA by establishing standardized criteria for AV differentiation in small-field (3 × 3 mm^2^) scans. We demonstrate that accurate and reproducible AV labeling is achievable within this limited field of view, supported by high inter- and intra-rater reliability. To overcome the lack of anatomical landmarks typically used in wide-field imaging, we introduce a NIR-based mapping strategy that aligns high-resolution OCTA with gold-standard CFP, enabling precise ground truth generation. Although intrinsic vessel features such as diameter and intensity showed limited discriminative power, our findings highlight the value of formalized OCTA AV differentiation criteria. Ultimately, this work lays a foundational framework for consistent manual labeling and future machine learning applications in macular OCTA, addressing a critical gap in macular small FOV OCTA image segmentation.

## Figures and Tables

**Table 1:** Assessment of morphological features for artery and vein differentiation.

Key differentiating feature	Description	Utility in macular AV differentiation	Applicability in practice
CFZ	Arteries are surrounded by a paCFZ, visible as a dark halo	Highly reliable	A primary and robust intra-image feature. Visibility and applicability persist even in small FOV images
Vessel crossings	Arteries do not cross other arteries nor do veins cross other veins; most vascular crossings occur between AV pairs	Limited in a small FOV	Crossings are uncommon at pseudo-first- and pseudo-second-order levels in small FOV scans and are absent at pseudo-third-order branches. However, when present, they provide strong visual confirmation for AV differentiation
Vessel caliber	Arteries typically appear thinner than adjacent veins	Not applicable in a small FOV	A reliable feature in wide-field OCTA and for large proximal vessels, where the arteries appear thinner than adjacent veins. However, its utility diminishes in macular FOV scans, where caliber differences diminish among distal branches
Vessel intensity	Arteries and veins exhibit different intensity profiles/brightness	Not suitable for manual differentiation	The difference is not perceptible to the human eye. However, machine learning models can extract and utilize these weak intensity differences for classification
Vessel tortuosity	Tortuosity quantifies vessel curvature. Arteries are typically straighter and veins more tortuous	Not suitable for manual differentiation, not applicable in a small FOV	In small-field OCTA (e.g., 3 × 3 mm^2^), vessel segments are shorter and often lack full context or curvature trajectories, which limits the reliability of tortuosity measurements making tortuosity less reliable for AV differentiation

**Table 2:** Accuracy of artery–vein differentiation.

	Annotator 1			Annotator 2		
	Accuracy 1	Accuracy 2	Avg accuracy	Accuracy 1	Accuracy 2	Avg accuracy
Total (465 vessels)	95.1% (23 errors)	95.1% (23 errors)	95.1%	92.0% (37 errors)	92.7% (34 errors)	92.3%
Pseudo-first order (315 vessels)	94.6% (17 errors)	94.9% (16 errors)	94.8%	92.4% (24 errors)	93.3% (21 errors)	92.9%
Pseudo-second order (115 vessels)	94.8% (6 errors)	93.9% (7 errors)	94.3%	90.4% (11 errors)	90.4% (11 errors)	90.4%
Pseudo-third order (35 vessels)	100% (0 errors)	100% (0 errors)	100%	94.3% (2 errors)	94.3% (2 errors)	94.3%

**Table 3:** Intra- and inter-rater reliability for artery–vein differentiation.

	Annotator 1 intra-rater reliability	Annotator 2 intra-rater reliability	Inter-rater reliability (Cohen’s *κ*)
Total (465 vessels)	0.99	0.98	0.84
First order (315 vessels)	0.99	0.97	0.82
Second order (115 vessels)	0.98	1	0.84
Third order (35 vessels)	1	1	0.80
